# Plasmonic gold nanostars for synergistic photoimmunotherapy to treat cancer

**DOI:** 10.1515/nanoph-2021-0237

**Published:** 2021-08-27

**Authors:** Yang Liu, Ericka Chorniak, Ren Odion, Wiguins Etienne, Smita K. Nair, Paolo Maccarini, Gregory M. Palmer, Brant A. Inman, Tuan Vo-Dinh

**Affiliations:** Department of Biomedical Engineering, Duke University, Durham, NC, 27708, USA; Department of Chemistry, Duke University, Durham, NC, 27708, USA; Fitzpatrick Institute of Photonics, Duke University, Durham, NC, 27708, USA; Department of Radiation Oncology, Duke University Medical Center, Durham, NC, 27710, USA; Division of Urology, Duke University Medical Center, Durham, NC, 27710, USA; Department of Surgery, Duke University Medical Center, Durham, NC, 27710, USA; Department of Pathology, Duke University Medical Center, Durham, NC, 27710, USA; Department of Neurosurgery, Duke University Medical Center, Durham, NC, 27710, USA; Department of Electrical and Computer Engineering, Duke University, Durham, NC, 27708, USA

**Keywords:** cancer, gold nanostars, photoimmunotherapy

## Abstract

Cancer is the second leading cause of death and there is an urgent need to improve cancer management. We have developed an innovative cancer therapy named Synergistic Immuno Photothermal Nanotherapy (SYMPHONY) by combining gold nanostars (GNS)-mediated photothermal ablation with checkpoint inhibitor immunotherapy. Our previous studies have demonstrated that SYMPHONY photoimmunotherapy not only treats the primary tumor but also dramatically amplifies anticancer immune responses in synergy with checkpoint blockade immunotherapy to treat remote and unresectable cancer metastasis. The SYMPHONY treatment also induces a ‘cancer vaccine’ effect leading to immunologic memory and prevents cancer recurrence in murine animal models. This manuscript provides an overview of our research activities on the SYMPHONY therapy with plasmonic GNS for cancer treatment.

## 1 Introduction

Cancer has been a severe threat to human health and 10 million people die of cancer worldwide in 2020 [1, 2]. Therefore, there is an urgent need to improve cancer management. Laser interstitial thermal therapy is an emerging the Food and Drug Administration (FDA)-approved treatment that uses a stereotactically-guided laser to ablate tumors mini-invasively [3]. While ablative thermal therapy (>55 °C) can actually induce immediate cell death in target tumors, mild fever-range thermal therapy (40–43 °C) can be used to (1) improve drug delivery to tumors, (2) improve cancer cell sensitivity to anticancer therapy, and (3) trigger potent systemic anticancer immune responses [4], [5], [6], [7]. Nanoparticle-mediated thermal therapy offers the potential to combine the advantages of precise cancer cell ablation with many benefits of mild thermal therapy in the tumor microenvironment, including radio-sensitization of hypoxic regions, enhancement of drug delivery, activation of thermosensitive agents, and boosting the immune system [8], [9], [10], [11].

Among various nanoparticles used for photon-induced thermal therapy, gold nanostars (GNS) are of particular interest as they offer a wide range of optical tunability by engineering subtle changes in their geometry [12], [13], [14], [15]. The multiple sharp branches on GNS create a “lightning rod” effect that enhances the local electromagnetic field dramatically and concentrates heating into cancer cells [16]. We can tune this unique tip-enhanced plasmonic property in the near-infrared (NIR) tissue optical window, where photons travel further in healthy tissue to be ‘captured’ and converted into heat by GNS within cancer [17]. Our team pioneered GNS development using a novel surfactant-free synthesis method for safe and effective star-shaped nanoparticle generation [18]. In contrast with traditional thermal therapy modalities, such as ultrasound, microwaves, and radiofrequency, GNS-photothermal therapy (PTT) can rapidly and precisely ablate tumor cells and induce mild hyperthermia in their microenvironment since GNS preferentially accumulate in tumors [19]. This enhanced permeability and retention (EPR) feature and the capacity to efficiently convert photon energy into heat, make GNS the ideal photothermal transducer for selective cancer therapy at the nanoscale level as demonstrated by our group in both *in vitro* and *in vivo* experiments [20].

Immune checkpoint blockade (ICB) is a revolutionary milestone in cancer treatment. ICB has been demonstrated to unleash cytotoxic T cells by reversing tumor immunosuppressive microenvironments to destroy cancer cells [21]. Among different ICB, anti-PD-1/PD-L1 immunotherapy is one of the most promising approaches [22]. Programmed death-ligand 1 (PD-L1), a protein overexpressed by many cancers (including brain tumors), binds to its receptor PD-1 on activated, infiltrating T cells, inhibiting their cytotoxic antitumor function, and facilitating immune escape [23], [24], [25], [26]. Therapeutic anti-PD-1/PD-L1 antibodies have been FDA approved for a variety of solid tumors treatment and generated survival improvement [27], [28], [29]. However, only a fraction of patients can benefit from ICB, urging for novel methods to improve immunotherapeutic response rate and efficacy [30]. Combining ICB with other therapies has been considered a promising approach to improve immunotherapeutic efficacy [31, 32]. Anti-PD-1 ICB has been combined with hollow gold nanoshell-enhanced photothermal therapy for cancer treatment [33]. The reported data demonstrated that the combination therapy could not only effectively eradicate the primary tumors but also inhibit the growth of metastatic tumors. Cytotoxic T lymphocyte antigen-4 (CTLA-4) is another promising immune checkpoint system and its inhibitor has been approved by FDA for human cancer treatment [34]. Anti-CTLA-4 immunotherapy has been combined with carbon nanotubes-based photothermal therapy to treat breast cancer [35]. Experimental results have demonstrated that the induced anticancer immune responses in combination with ICB treatment could effectively treat secondary tumors and lung metastasis. Another study combined anti-CTLA-4 immunotherapy with Prussian blue nanoparticles-based photothermal therapy to treat neuroblastoma, which is a common and treatment-resistant pediatric cancer [36]. The results of this study showed that the combined treatment was more durable against neuroblastoma and the long-term survivors could reject cancer rechallenge, indicating the existence of memorized anticancer immune responses.

We have developed a novel cancer photoimmunotherapy by synergistically combining GNS-enhanced photothermal therapy with checkpoint blockade immunotherapy, henceforth named SYnergistic iMmuno PHOtothermal NanotherapY (SYMPHONY). The developed SYMPHONY therapy has been demonstrated to trigger memorized anti-cancer immune responses to treat not only cancer metastasis but also prevent cancer recurrence [37, 38]. This manuscript provides an overview of our research activities on the development of SYMPHONY photoimmunotherapy with plasmonic GNS for cancer treatment.

## 2 Plasmonic GNSs for photoimmunotherapy

### 2.1 Synthesis and characterization of plasmonic GNSs

**Figure 1:**
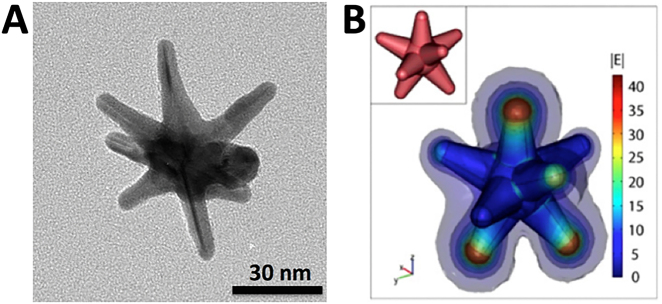
(A) Transmission electron microscopy (TEM) image of the synthesized GNS nanoprobe. (B) Electromagnetic field simulation shows significantly enhanced electromagnetic field at GNS tips. (Adapted from Ref [18, 38]).

Our team has first introduced a novel surfactant-free synthesis method, not requiring toxic cetyltrimethylammonium bromide (CTAB), to produce star-shaped gold nanoparticles that are biocompatible for *in vivo* applications [18]. Briefly, 12-nm spherical gold nanoparticles synthesized by reducing HAuCl_4_ with trisodium citrate are used as seeds. For GNS synthesis, AgNO_3_ (0.1 ml, 3 mM) and ascorbic acid (0.05 ml, 100 mM) were simultaneously added to 10 ml of water solution with 0.25 mM HAuCl_4_, 1 mM HCl, and 0.2 ml of 12-nm gold sphere nanoparticles under vigorous stirring. The synthesized GNS are functionalized with thiolated polyethylene glycol (Thiol-PEG, M.W. 6000) by incubating at room temperature for 24 h to improve *in vivo* stability and circulation time. The synthesized GNS are unique among different metallic nanoparticles due to their extensively branched geometry as shown in the TEM image in Figure 1(A). Under laser excitation, the surface of the metallic nanoparticle manifests a large electromagnetic field enhancement and creates local hotspots where there is a curvature on the surface charge density. GNS’ branched shape induces the lightning-rod effect magnifying the electromagnetic field dramatically at the tips where the curvature is greatest as shown in the electromagnetic field simulation (Figure 1(B)). This tip-enhanced plasmonic effect leads to the high photon to heat conversion efficiency that allows it to be effective for cancer photothermal therapy.

### 2.2 GNS-enhanced photothermal study in phantom models

**Figure 2:**
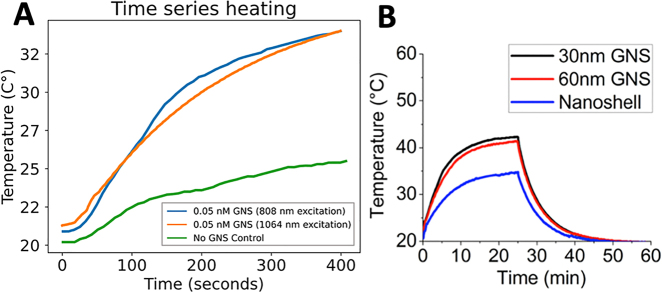
(A) Heating comparison of a phantom with and without GNS under 808 and 1064 nm laser irradiation. The laser power density was set to be 0.6 W/cm^2^ and a thermocouple probe was used to measure the temperature at 1-s intervals. (B) Heating comparison of GNS with gold nanoshells under 808 nm laser irradiation. (Adapted from Ref [19]).

Traditional laser ablation therapy has the limitations of small treatable lesions and a lack of specific conformity to tumor margins. Both shortcomings can be potentially addressed by using plasmonic GNS with high photon-to-heat conversion efficiency. We have used phantom models to demonstrate that GNS can dramatically enhance photothermal treatment with near-infrared laser in the tissue “optical window” range of 700–1100 nm, where light is minimally absorbed by the tissue. The plasmonic peak of GNS can be easily tuned in the near-infrared region by changing the synthesis procedure to have high absorbance in the selected laser wavelength for *in vivo* photothermal therapy. Figure 2(A) shows GNS can markedly increase heating rates with near-infrared laser within the phantom even at a low GNS’s concentration of only 0.05 nM. In addition, we have compared GNS with gold nanoshells for photothermal conversion. Figure 2(B) shows that GNS have a much higher equilibrium temperature (42.3 and 41.4 °C) than nanoshells (34.7 °C) at the equivalent optical density. The calculated values of photothermal conversion efficiency were 94% for 30-nm GNS, 90% for 60-nm GNS, and 61% for gold nanoshells [19]. This study result demonstrates that GNS have a higher efficiency in converting the laser photons into heat than gold nanoshells, which are in a clinical trial for cancer photothermal therapy.

### 2.3 *In vivo* study of GNS-enhanced photothermal therapy

**Figure 3:**
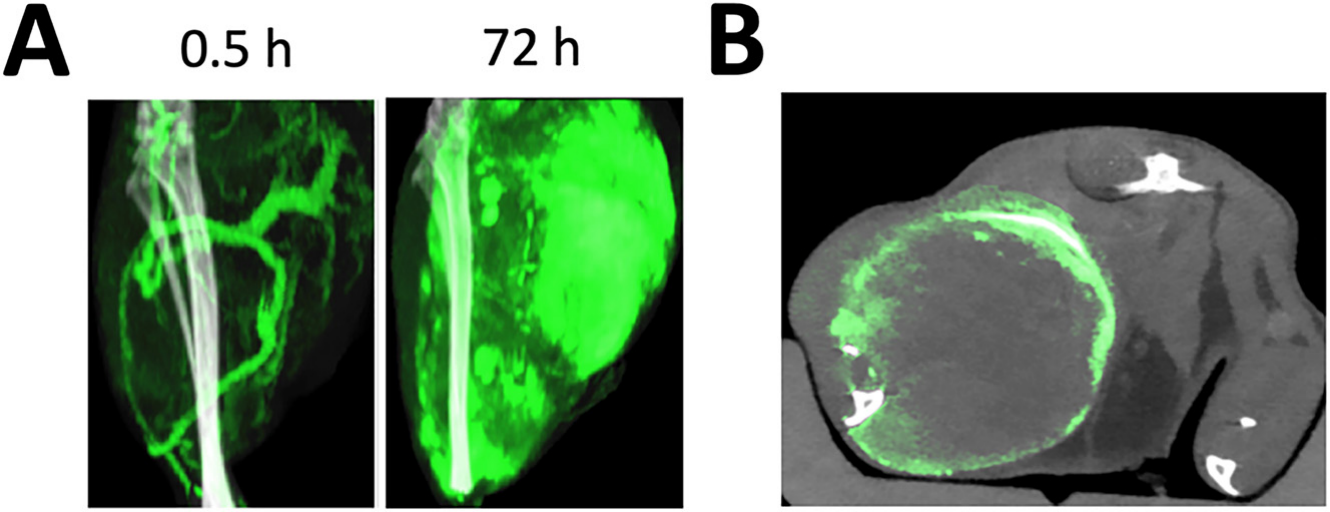
(A) Maximum intensity projection of dual-energy CT images for the tumor in the hind leg 0.5 and 72 h after intravenous injection of GNS. The green color shows GNS concentration in the range of 2–10 mg/ml and the gray color shows tissues windowed from −100 to 5000 HU. (B) Dual-energy CT image for tumor 72 h after GNS intravenous injection. The green color shows GNS in the concentration range from 2.5 to 8 mg/ml and the gray color shows tissues windowed from −500 to 1200 HU. (Adapted from Ref [19]).

**Figure 4:**
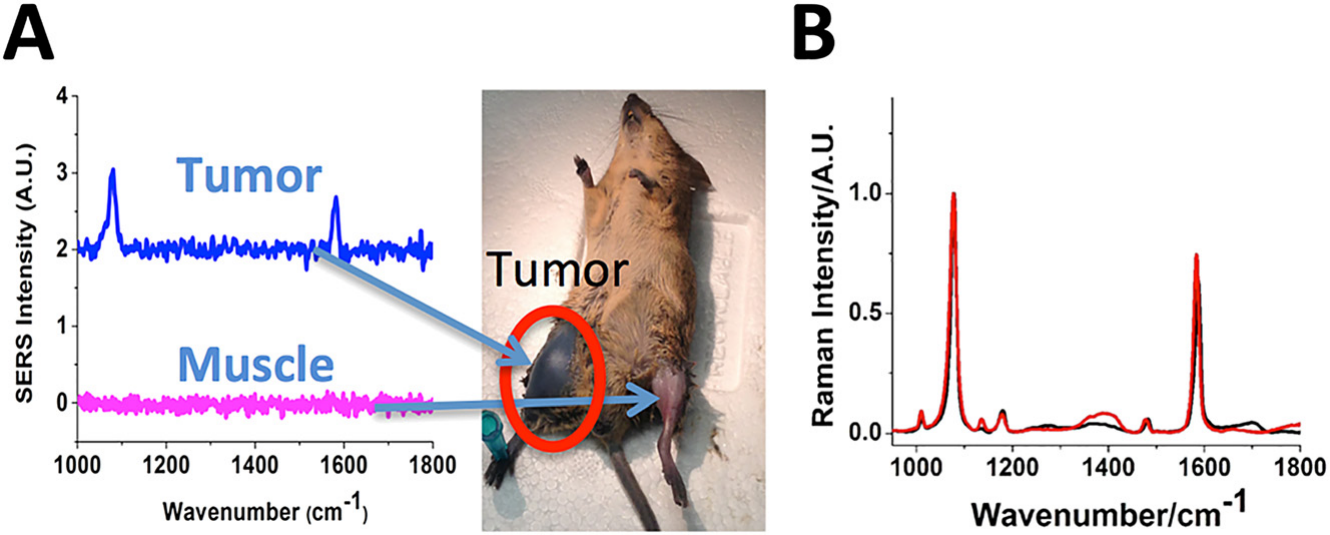
(A) *In vivo* SERS spectrum measured in the tumor and healthy muscle 72 h after intravenous injection of GNS SERS nanoprobe. Unique SERS peaks can be detected in the tumor but not healthy muscle. The tumor shows black color due to a high uptake of GNS which absorbs light strongly. (B) SERS spectrum of GNS nanoprobe in the solution at pH 5 (black) and pH 9 (red). (Adapted from Ref [19, 39]).

We have demonstrated that GNS can selectively accumulate in the tumor for photothermal therapy using longitudinal dual-energy computed tomography (CT) imaging and a murine sarcoma animal model [19]. Figure 3(A) shows GNS distribution at various time points after systemic administration through tail vein. GNS were found to be mainly in blood vessels 30 min after intravenous injection. It appears that the majority of blood vessels in the tumor region were located in the tumor rim. As a result, perfusion to the tumor core may be limited due to a lack of blood flow. At the 72-h time point, a significant amount of GNS was located in the tumor. A CT slice through the tumor shows a heterogeneous intratumoral distribution of GNS and the GNS’s concentration at the rim is much higher than that in the center (Figure 3(B)). The low GNS concentration at the tumor center may reflect poor perfusion in the tumor core. Furthermore, we have performed *in vivo* surface-enhanced Raman spectroscopy (SERS) to confirm that GNS can selectively accumulate in the tumor. As shown in Figure 4(A), the SERS signal of GNS with p-mercaptobenzoic acid can be detected in the tumor but not the healthy tissue. We have also performed a theoretical calculation to uncover the vibrational modes of detected SERS peaks from *in vivo* Raman measurement [39]. The detected SERS peak at 1077 cm^−1^ was found to be due to the benzene ring breathing mode and the SERS peak at 1588 cm^−1^ was due to the benzene ring stretching mode. Our study results demonstrate that GNS can selectively accumulate in the tumor for photothermal therapy.

**Figure 5:**
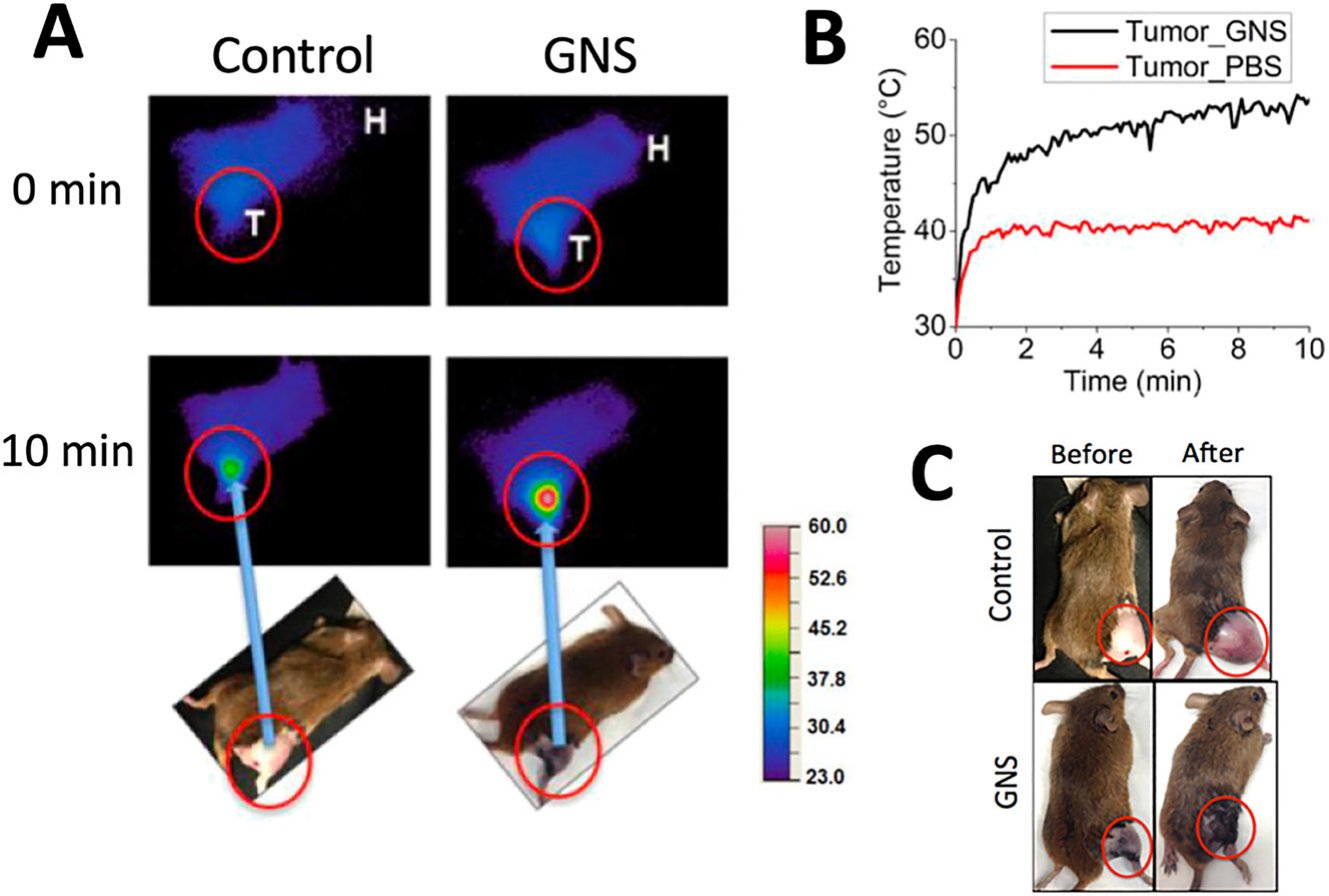
(A) Thermal imaging with a near-infrared camera to monitor tumor temperature change under laser irradiation with and without GNS. The temperate scale bar is from 23 to 60 °C. T shows tumor location and H represents the mouse head. (B) Tumor temperature change profile for the mouse with IV injection of GNS or phosphate-buffered saline (control). (C) Photographs of mice before and after laser treatment with and without GNS injection. The tumor size for the mouse with both GNS injection and laser irradiation had a clear tumor size decrease while the tumor for the mouse with only laser irradiation grew quickly. (Adapted from Ref [19]).

After demonstrating GNS’ high photon-to-heat conversion efficiency and selective accumulation in the tumor, we have performed *in vivo* photothermal therapy to demonstrate that near-infrared (NIR) laser with GNS can ablate the tumor effectively [19]. The tumor temperature during laser treatment was monitored by using an infrared thermal imaging camera (Thermo Tracer TS 7302, NEC, Japan). As shown in the Figure 5(A) and (B), NIR thermal imaging results show that the tumor temperature for the mouse with GNS administration is significantly higher than that for the mouse tumor without GNS under the same laser irradiation. A combination of GNS and NIR laser can increase tumor temperature to be more than 50 °C, which is in the high-temperature hyperthermia range for ablation. The tumor treated with both GNS and laser irradiation regressed dramatically while the tumor treated with only laser irradiation grew quickly. In addition, the ablation effect for GNS-enhanced photothermal therapy was confined primarily in the tumor and there was no detectable tissue damage outside of the tumor region. Although there was some skin burning directly over the tumor surface, no other adverse effects were observed in these mice. *In vivo* study results with the murine animal model demonstrate that GNS-mediated photothermal therapy can ablate tumors efficiently and specifically.

### 2.4 GNS-enhanced SYMPHONY therapy for cancer treatment

**Figure 6:**
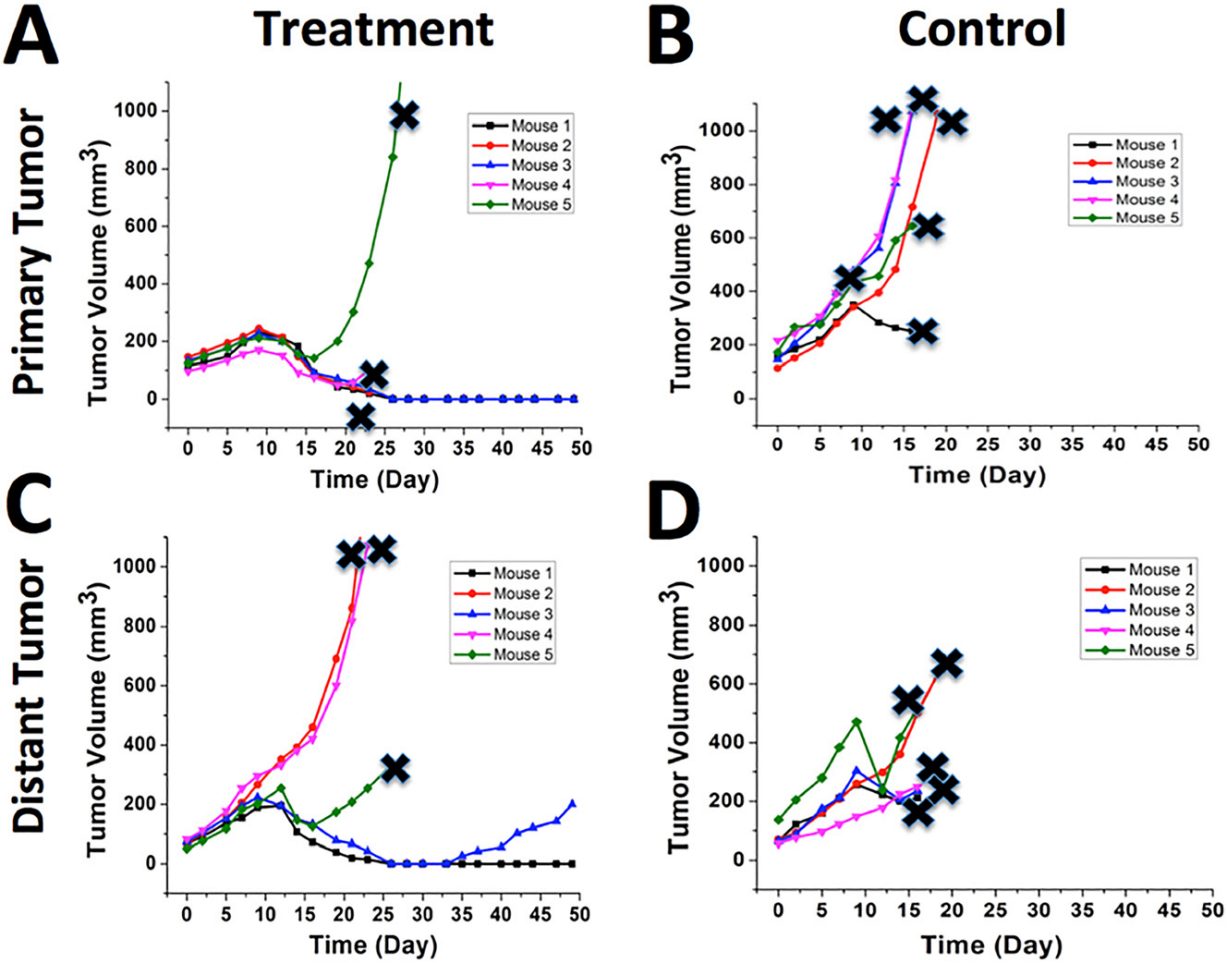
Primary tumor size changes profile for the mice with SYMPHONY treatment (A) and blank control (B). Distant tumor size changes profile for the mice with SYMPHONY treatment (C) and blank control (D). The laser irradiation of photothermal therapy was performed only on the primary tumor. The line stopped (× sign in black color) if the mouse was sacrificed due to a large tumor or ulceration. (Adapted from Ref [37]).

**Figure 7:**
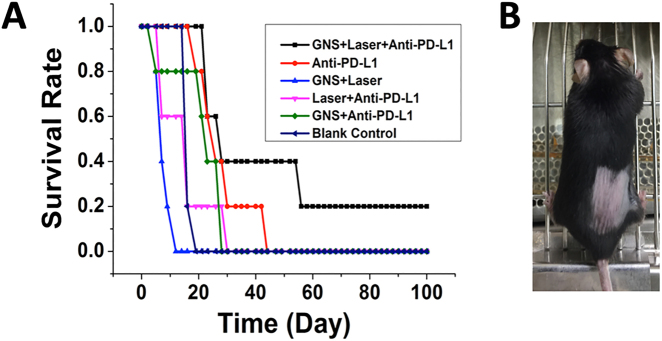
(A) Kaplan–Meier (K-M) overall survival curve. At the end of 49 days, only the SYMPHONY (GNS + Laser + Anti PDL1) group had two surviving mice (40%) and only this group had one mouse that survived after 100 days; all other control groups have no survival mice. (B) For the tumor-free mouse from SYMPHONY therapy, no tumor developed after rechallenge indicating the existence of memorized anticancer immune responses. (Adapted from Ref [37]).

Bladder cancer with a high recurrence rate is a major public health issue and it is the fourth most common cancer in men [40], [41], [42], [43], [44], [45]. There is a clinical need for novel and effective approaches to managing bladder cancer that reduce tumor recurrences and treatment costs, while improving patient life duration and quality. We have demonstrated that a combination of GNS-enhanced photothermal therapy and checkpoint blockade immunotherapy, SYMPHONY therapy, can be far superior to any of its individual components using a murine bladder cancer animal model [37]. We used a dual flank tumor model with MB49 bladder cancer cell line and C57BL/6 laboratory mice to demonstrate that SYMPHONY therapy could treat not only the primary tumor, but also trigger anticancer immune responses to treat cancer metastasis and prevent cancer recurrence. Figure 6(A) shows that the treated primary tumor after SYMPHONY photoimmunotherapy shrank starting on Day 8. Two mice exhibited no measurable tumors from Day 26 until Day 49. Two mice had tumor sizes that decreased but were sacrificed due to the large tumors on the other side. One mouse had tumor sizes that decreased first but then increased after approximately one week. GNS-enhanced photothermal therapy not only generates an immediate therapeutic effect at the tumor with laser ablation but also induces anticancer immune responses. As shown in Figure 6(C), the distant tumor (used as a model of cancer metastasis) also got treated after SYMPHONY therapy. Two of five mice had both tumors disappear between Day 25 and Day 33. One surviving mouse was tumor-free three months after SYMPHONY therapy and had no tumor development even after rechallenge, indicating the existence of a memorized anti-cancer immune response (Figure 7). The observed synergistic therapeutic effect could be due to the generation of anticancer immune responses as the result of the GNS-mediated photothermal therapy, which causes the release of cancer-specific antigens. It has been reported that hyperthermia can trigger anticancer immune responses and generate cancer-specific cytotoxic T cells by exposing the calreticulin (CALR) on the dying cell surface and extracellularly releasing damage-associated molecular patterns (DAMP) including heat shock proteins (HSP), adenosine 5-triphosphate (ATP), and high-mobility group box 1 (HMGB1); these species actively participate in the immune response by modulating maturation, activation, and presentation of antigen-presenting cells (APCs) [5, 46, 47]. The generated anti-cancer immune cells can be further enhanced by reversing tumor immunosuppressive microenvironment with checkpoint inhibitor immunotherapy with anti-PD-L1 antibody. Given that a laser can be introduced intravesically via a cystoscope and the US FDA has approved both laser interstitial thermal therapy and PD-L1 based immunotherapy, the SYMPHONY therapy could provide an effective treatment when an aggressive tumor cannot be surgically resected. This strategy could represent a next-generation treatment paradigm that challenges traditional surgical resection for bladder cancer.

## 3 Conclusion and future perspective

In summary, we have demonstrated that the plasmonic GNS-enhanced photoimmunotherapy can not only treat the primary tumor but also trigger systemic anticancer immune responses to treat cancer metastasis, which is better than any single treatment modality tested here. With their unique plasmonic properties, GNS can amplify the optical properties of the laser light and thus increase the effectiveness of light-based photothermal treatment. The combination of targeted nanoparticle-enabled photothermal therapy can improve checkpoint blockade immunotherapy. The effectiveness of the combination of photothermal nanotherapy and PD-L1 immunomodulation was demonstrated to be synergistic (not just additive) and delayed rechallenge with cancer cells injection did not lead to new tumor formation, indicating that the combined treatment induced effective long-lasting immunity against cancer and prevent cancer recurrence like an “anticancer vaccine”.

The GNS contribute to plasmonics-amplified immune nanotherapy by exhibiting multiple unique features: (i) they are effective plasmonic nano enhancers of light, (ii) they can be used for nanotargeting tumor cells, (iii) they are nanosources for heating tumor cells from the inside, and (iv) they play the role of nanoactivators of the immune system. The therapeutic efficacy of the SYMPHONY therapy could be further enhanced by optimizing treatment parameters such as laser wavelength, laser dose, treatment sequence, and checkpoint inhibitor delivery doses and regiments. Future fundamental studies on mechanistic understanding of induced anticancer immune responses by plasmonic nanoparticles-mediated photothermal therapy would be of great importance for its further development and clinical translation. The optimized SYMPHONY therapy could also be applied to treat other aggressive cancer diseases such as melanoma and breast cancer aimed for clinical translation to substantially improve outcomes of cancer patients, especially those that are currently ineligible or unresponsive to the standard of care treatment.
